# Gravure Printing of Water-based Silver Nanowire ink on Plastic Substrate for Flexible Electronics

**DOI:** 10.1038/s41598-018-33494-9

**Published:** 2018-10-11

**Authors:** Qijin Huang, Yong Zhu

**Affiliations:** Department of Mechanical and Aerospace Engineering, North Carolina State University, Raleigh, North Carolina 27695-7910 USA

## Abstract

Gravure printing is a promising technique for large-scale printed electronics. However, gravure printing of silver nanowires (AgNWs) so far has been limited in terms of resolution and electrical conductivity. In this study, gravure printing of water-based AgNW ink on a flexible substrate is demonstrated. By tailoring the ink properties, printing conditions and post-printing treatment, gravure printing enables printing of high-resolution, highly conductive AgNW patterns in large areas, with resolution as fine as 50 µm and conductivity as high as 5.34 × 10^4^ S cm^−1^. The printed AgNW patterns on the flexible substrate show excellent flexibility under repeated bending. All these characteristics demonstrate the excellent potential of gravure printing of AgNWs for developing large-area flexible electronics.

## Introduction

Printed electronics has drawn tremendous interest in the past few decades^1–6^, as it offers an attractive alternative to conventional silicon-based fabrication technologies by enabling low-cost, large-area, flexible devices for many applications such as energy storage^7^, thin film transistor^8^, light-emitting diodes^9^ and wearable sensors for health monitoring^10^. Central to this technology are high-performance functional inks and high-throughput printing methods, such as screen^11–15^, inkjet^16–18^, gravure^19,20^ and flexographic printing^21^. Among the existing printing methods, gravure printing, which utilizes direct transfer of functional inks through physical contact of engraved structures with a substrate, is a promising option for large-scale applications due to its high-speed, high-resolution deposition of functional materials and compatibility with roll-to-roll processes.

One important application of gravure printing is the fabrication of conductive elements as electrodes and conductors. The scope of gravure-printed electronic materials has been previously limited to polymers (*e.g*. PEDOT:PSS)^22^ and nanoparticles^23,24^. Recently, one-dimensional nanomaterials (*e.g*. metal nanowires^25,26^ and carbon nanotubes^27,28^) and two-dimensional nanomaterials (*e.g*. graphene)^29,30^ have been gravure-printed on flexible substrates as flexible and transparent electrodes and interconnects. Among these nanomaterials, silver nanowires (AgNWs) have emerged with promising potential in electronic applications^31–43^. Compared to the silver particles used in the traditional silver inks, AgNWs offer better electrical conductivity and flexibility, which are key to flexible electronics applications. There have been recent studies on gravure printing of AgNWs as transparent conductive films^25,26^, However, the best reported resolution was limited to 230 µm^25^. The printing resolution of gravure printing is mainly dependent on ink properties (*e.g*. surface tension and viscosity)^44^ in addition to trench resolution^24^. It remains challenging to realize high-resolution gravure printing of AgNWs as a result of their large length-to-diameter aspect ratio.

Here we report large-scale, high-resolution patterning of AgNWs by gravure printing. A new type of water-based AgNW ink was developed. Rheological behavior of the ink was investigated to correlate the ink compositions, the rheological properties and the printing results. By tailoring the ink properties and printing conditions, continuous lines with resolution as fine as ~50 µm were achieved over large areas with notable reliability and uniformity. The conductivity of the printed AgNW lines was measured to be as high as 5.34 × 10^4^ S cm^−1^. In addition, the printed AgNW lines on a flexible polyethylene terephthalate (PET) film showed excellent flexibility under repeated bending.

## Results

**Figure 1:**
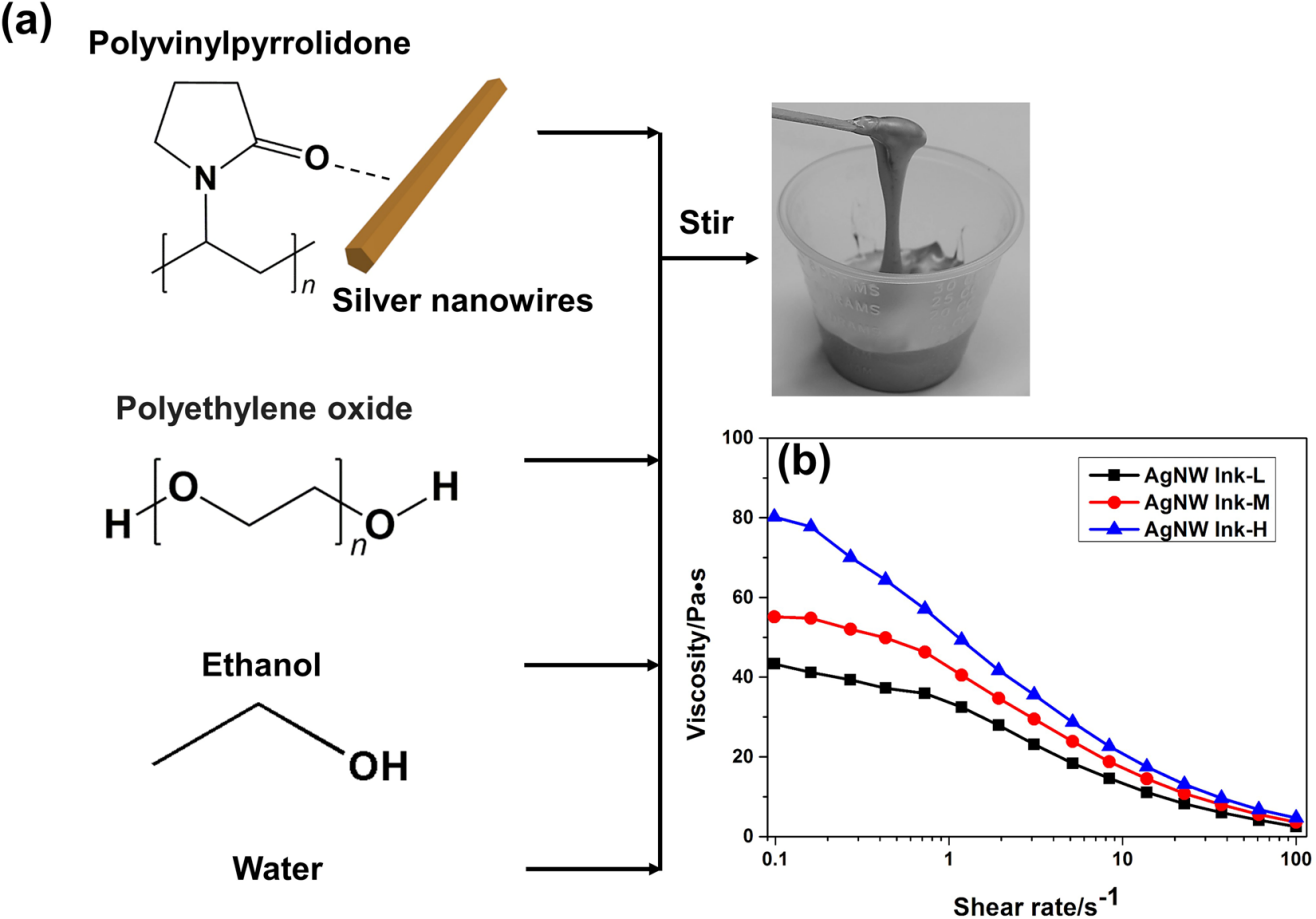
(**a**) Fabrication process of AgNW ink and photograph of the 5 wt% AgNW ink. (**b**) Viscosity as a function of shear rate for different concentration of AgNW inks.

The AgNWs synthesized by the modified polyol reduction method were characterized by SEM and TEM, as shown in Fig. S1a. The AgNWs present a relatively high aspect ratio (~200) with an average diameter of 80 nm and average length of 15 μm. Fig. S1b shows the XRD pattern of dried AgNWs. The four strongest peaks can be observed at 38.2°, 44.4°, 64.5° and 77.5°, which were attributed to the diffraction of the (111), (200), (220) and (311) crystalline planes of the face-centered structure of silver, respectively, according to the Silver Joint Committee on Powder Diffraction Standards Database (JCPDS, File No. 04-0783). The printed conductor consists of AgNWs in the form of a percolation network. To formulate the AgNW ink for gravure printing, several properties, including stabilization of AgNWs in the ink and wetting, spreading, adhesion and drying of printed AgNW patterns, should be optimized. These properties are crucial for the fabrication of high-resolution, uniform AgNW patterns. The ink was prepared by adding AgNWs to a viscous poly(ethylene oxide) (PEO) solution with DI water and ethanol as co-solvents (Fig. 1a). DI water was chosen as the major solvent due to its environmental friendliness and low cost. Due to the high surface tension of DI water (72.8 mN•m^−1^ at 20 °C), DI water alone is usually undesirable, which tends to result in aggregation of AgNWs as a result of contact line recession and dewetting during evaporation. Thus, nontoxic ethanol with low boiling point and a lower surface tension (22.5 mN•m^−1^ at 20 °C) was added.

Viscosity is the most important ink parameter to tailor. PEO, a flexible, non-ionic water-soluble polymer used in a wide variety of applications, was used to assist ink formulation for the following reasons^45,46^. First, PEO has a high molecular weight (average Mv ~1,000,000), which can increase the viscosity of the ink dramatically, and provides thixotropic behavior for the AgNW ink; second, PEO can function as a dispersive agent to improve dispersion of AgNWs as the hydroxy groups can bond with the surface of the AgNWs; third, PEO is also alcohol-soluble and can precipitate together with AgNWs to generate solid composite sediments with good redispersion capability, which is conducive to forming a uniform, continuous pattern. In a typical formulation, DI water, ethanol and PEO were mixed by stirring at a weight ratio of 12.5:11.5:1 for 24 h to make homogeneous solution. Then, AgNWs were added into the solution to make the gravure-printing inks with three different AgNW solid contents: 3.0 wt% (AgNW Ink-L), 3.7 wt% (AgNW Ink-M) and 5.0 wt% (AgNW Ink-H). The inks were stirred for 10 min to obtain the final stable AgNW inks. As-prepared viscous AgNW Ink-H is shown in Fig. 1a. The surface tensions of the three inks were 32.6, 30.2 and 28.8 mN•m^−1^, respectively.

The rheological behavior of the AgNW inks was investigated to determine how the AgNW solid content affected the ink properties. Figure 1b shows that all three inks exhibited a shear thinning thixotropic behavior – viscosities of the inks decrease as the shear rate increases. At the same shear rate the AgNW ink with higher solid content exhibited higher viscosity. For example, the viscosities at the shear rate of 10 s^−1^ for the AgNW Ink-L, AgNW Ink-M, and AgNW Ink-H were 13.4, 16.9 and 20.9 Pa•s, respectively. This is because nanowires can act as an active cross-linker, leading to enhanced network strength and hence increased viscosity. The capillary number (*C*_*a*_) is a dimensional quantity to describe the relative effect of viscosity versus surface tension. *C*_*a*_ is defined as

$${C}_{a}=\frac{\eta U}{\gamma }$$

where *η* is the ink’s viscosity, *γ* is the ink’s surface tension and *U* is the printing speed (~1.5 mm•s^−1^ in this work). After calculation, the *C*_*a*_ for the AgNW Ink-L, AgNW Ink-M, and AgNW Ink-H were 0.62, 0.84, and 1.09, respectively. According to ref.^20^, at very low capillary number (*C*_a_ < 1), pattern fidelity was deteriorated by ink drag-out from the cells; at very high capillary number (*C*_a_ > 1), inefficient doctoring left ink in non-patterned areas. Optimal printing can be achieved by adjusting the printing speed and the ink parameters to make *C*_*a*_ ≈ 1^20^. Accordingly, the ink with a concentration of 5.0 wt% (AgNW Ink-H) was selected for printing in the rest of this work, as it possessed the rheological properties that best meet the printability.

**Figure 2:**
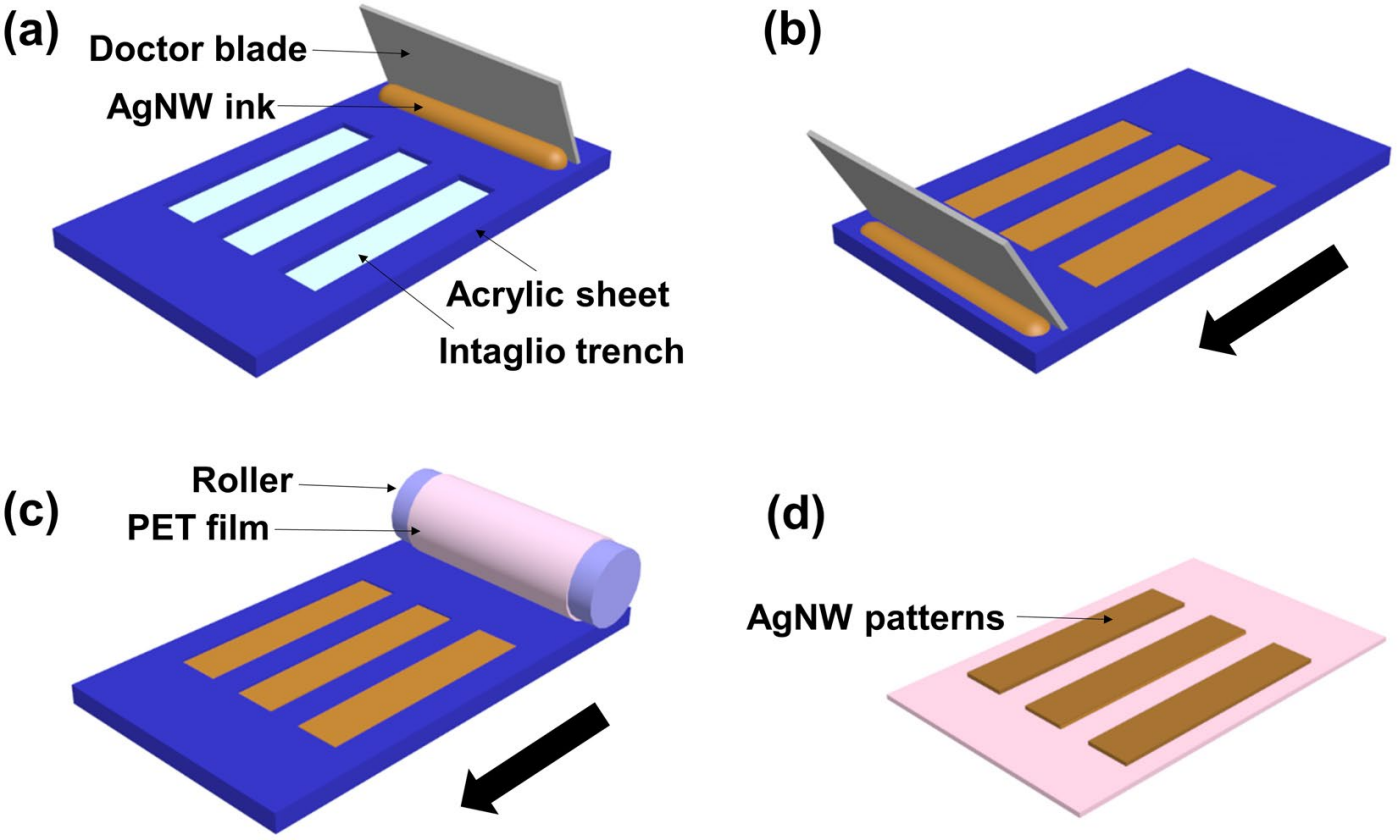
Schematic illusion of the inverse direct gravure printing process.

Inverse direct gravure printing was used to print AgNW patterns, as shown schematically in Fig. 2a–d. The printing process includes four steps: (1) applying ink to the gravure plate; (2) filling ink in the gravure trenches while removing the excess ink by a doctor blade; (3) transferring ink onto a flexible substrate on the roller; and (4) releasing the substrate from the roller. Acrylic sheet was used as the gravure plate in our work, and the trenches were fabricated by laser engraving. Trenches with different widths were obtained by adjusting the laser power. Unlike engraved cells used for printing silver nanoparticle inks^24^ and graphene inks^29^, long, continuous intaglio trenches^47^ were fabricated for the AgNW inks due to the large length of AgNWs. In our printing process, as shown in Fig. 2b, the ink filling and removing were operated at the same time by a doctor blade. A flexible PET film was mounted onto the roller and the ink was transferred onto the PET film as the roller rolled across the acrylic sheet (Fig. 2c). After releasing from the roller, gravure-printed conductive AgNW patterns were fabricated on the PET film (Fig. 2d).

## Discussion

**Figure 3:**
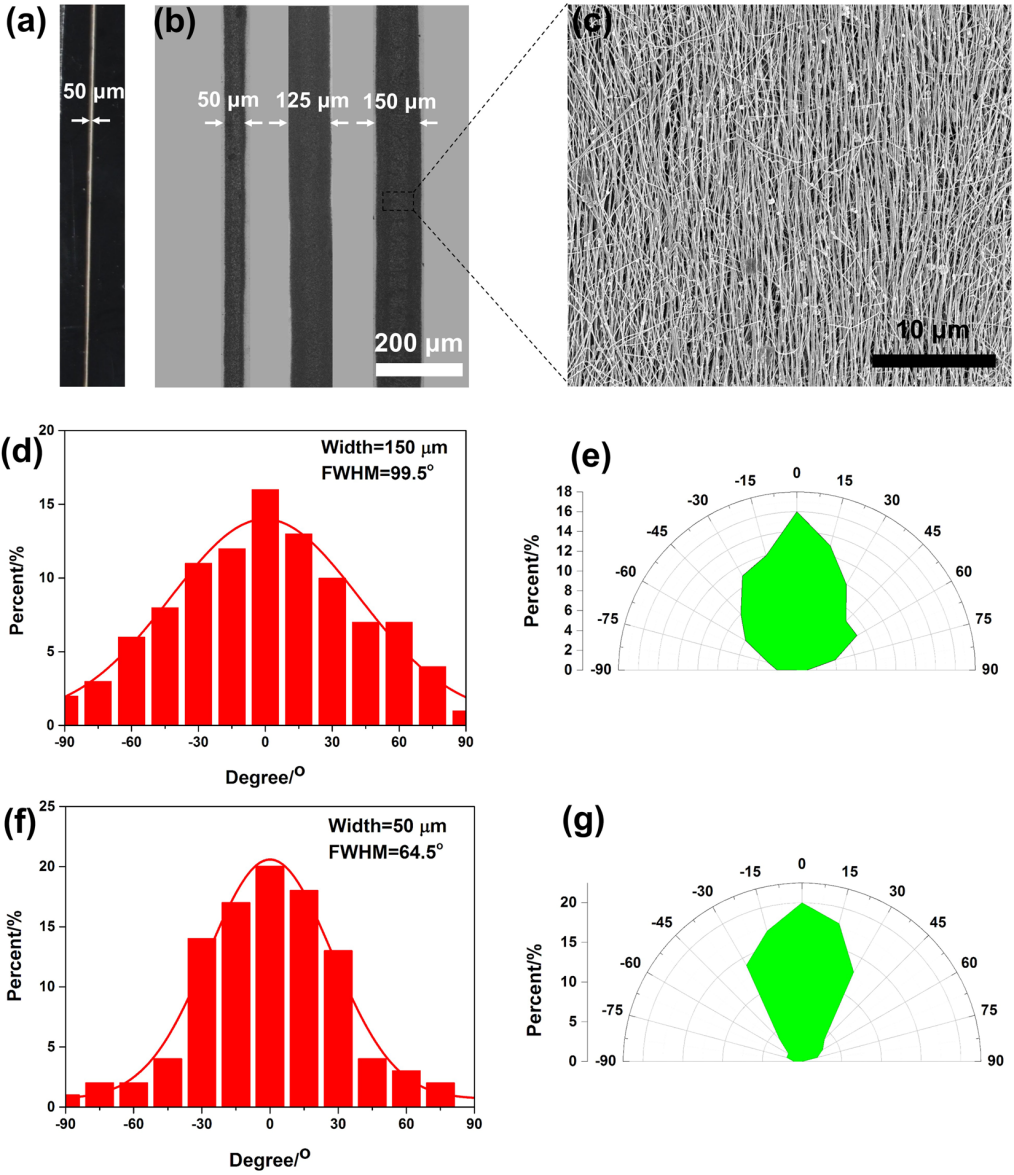
(**a**) Photograph of a printed AgNW line with length of 3 cm and width of 50 μm. (**b**) Optical image of three AgNW lines with different line widths (50, 125 and 150 μm) and the same spacing of 100 μm. (**c**) Morphology of the printed AgNW line. (**d**) and (**e**) Histograms and polar diagrams of oriented angles of the gravure-printed AgNWs lines with 150 μm line widths. (**f**) and (**g**) Histograms and polar diagrams of oriented angles of the gravure-printed AgNWs lines with 50 μm line widths.

Figure 3a shows the photograph of a gravure printed AgNW line with length of 3 cm and width of 50 μm. The AgNW line appeared to be continuous with uniform width and no voids. Figure 3b also shows an optical image of three AgNW lines with different widths (50, 100 and 125 μm) and the same spacing of 100 μm. Moreover, Fig. S2 shows an optical image of three AgNW lines with the same width of 50 μm and the same spacing of 100 μm. The edge of these lines were smooth and uniform. Further decreasing the line width or spacing would cause non-uniformity of the printed patterns. Thus, the printed resolution of line width and spacing between the lines can achieve 50 μm and 100 μm, respectively. During printing the doctor blade introduces shearing force to the AgNWs, which can align the AgNWs to the printing direction especially for those near the edges of the printed lines. This is similar to the case of patterning and alignment of AgNWs by manipulating wetting of dispersions in microchannels^48^. The printed AgNWs formed a dense percolation network and showed good alignment to the printing direction (Figs 3c and S3). The alignment was found to depend on the line width. Figure 3d,f show the distribution of the AgNW angles with respect to the printing direction for line widths of 150 µm and 50 µm, respectively. The AgNWs are better aligned along the printing direction for smaller line width. Full width at half-maximum (FWHM) values are also shown in Fig. 3d,f. As AgNW line width decreases from 150 µm to 50 µm, FWHM decreases from 99.5° to 64.5°. To better visualize the distribution of alignment of AgNWs, we plot the histograms into polar diagrams. As shown in Fig. 3e,g, the distribution of orientation angles starts from being a rather wide, radiative shape to being a narrow, ellipse shape with AgNW line width decreasing from 150 µm to 50 µm. Better alignment of AgNWs may contribute to improving electrical conductivity of the printed AgNW patterns^48,49^.

**Figure 4:**
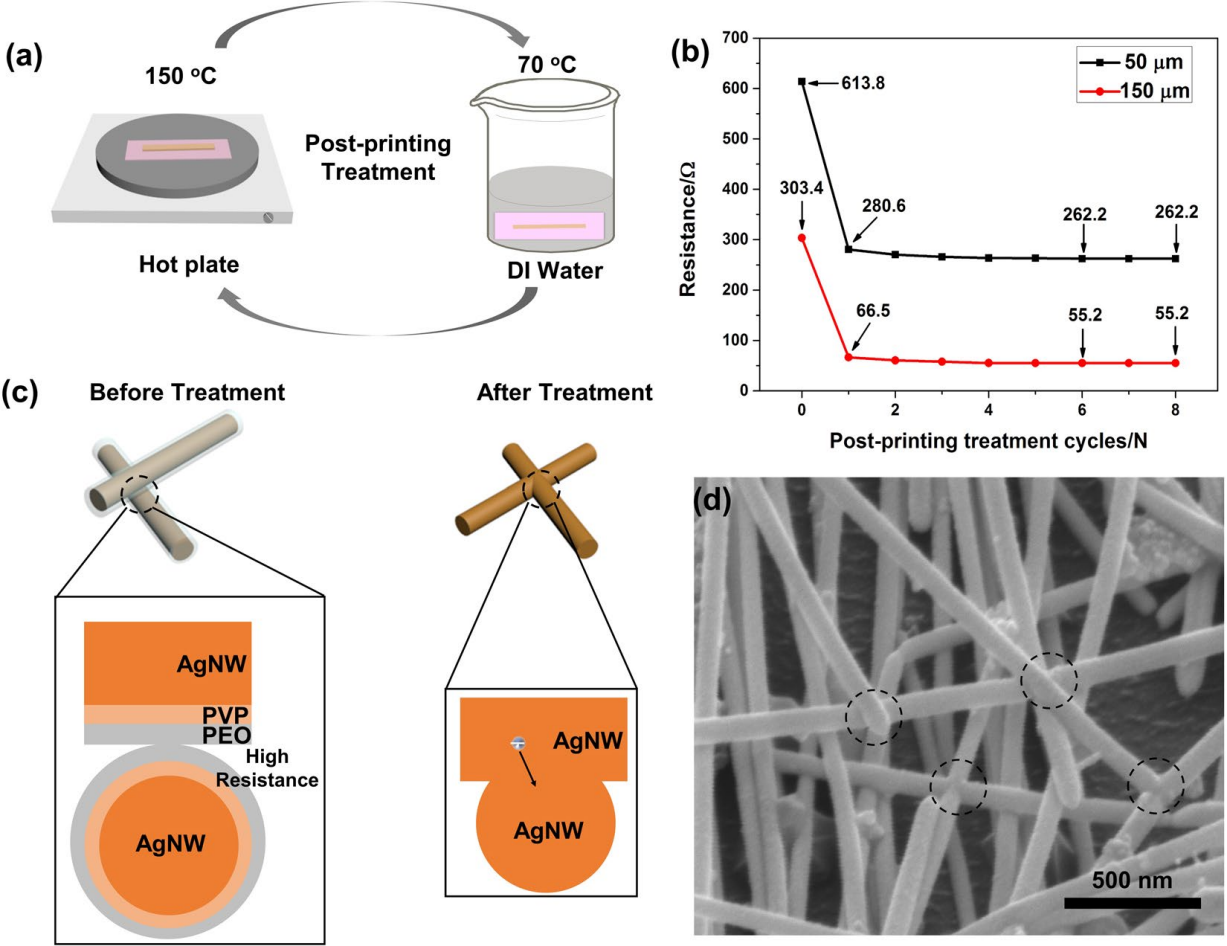
(**a**) Schematic illustration of the post-printing treatment process. (**b**) The resistance changes upon the different treatment cycles. (**c**) Schematic illustration of the mechanism of post-printing treatment. (**d**) High resolution SEM image of the line after post-printing treatment cycles of six.

Two types of water-soluble polymers existed in the AgNW inks - poly(vinylpyrrolidone) (PVP) coating that was introduced to control the growth of AgNWs during the synthesis (also serving as a surfactant that helps disperse AgNWs in solutions) and PEO that was used as the additive for formulating the AgNW inks. These non-conductive polymers can be seen as barriers to electron transport, so the printed AgNWs had relatively high electrical resistance. After gravure printing, post-printing treatment of the AgNW patterns, including thermal annealing and water washing, was developed to improve the electrical conductivity. Figure 4a shows the treatment cycles between thermal annealing (i.e. fusing the AgNW junctions at relative low temperature and not degrading the PET film, 150 °C for 2 min) and washing of the polymers (both PVP and PEO) in warm DI water (70 °C for 10 min). PVP glass transition temperature is around 150 °C. Thus heating at 150 °C, the mobility of PVP will significantly increase, which, when combined with water washing, will lead to their removal from the surface of the AgNWs and the junctions^50^, allowing a more intimate contact between AgNWs and thus a better electrical conduction of the network. Moreover, during water washing process, water soluble PVP and PEO can also be removed. The water washing process can further dissolve the PVP and PEO in the AgNW patterns. Thus, the post-printing treatment time and temperature are effective to remove most of the PVP. To obtain final AgNW patterns, the post-printing treated AgNW patterns were annealed at 150 °C for 5 min. Both steps are compatible with the polymeric substrate. To determine the number of treatment cycles, two typical lines with line width of 50 μm and 150 μm were gravure-printed on a PET film. For both lines electrical resistance was measured as a function of the number of treatment cycles, as shown in Fig. 4b. It can be seen that even for different line widths, the trend of the resistance change was nearly the same: the resistance decreased sharply after the first treatment cycle and then gently with increasing number of cycles until the sixth cycle, after which the resistance remained constant. The resistance drop was attributed to the removal of PVP and PEO from the AgNW lines, which apparently happened most dramatically during the first cycle. PVP and PEO were nearly completely removed after the sixth cycle. Thus, the number of treatment cycles used in this work was six. Interestingly, the height of the 125 μm-width AgNW line decreased from ~2 μm to ~1 μm after the six treatment cycles (Fig. S4). Figs S5 and 3c show the SEM images of the AgNW lines before and after the post-printing treatment, respectively.

The mechanism of the post-printing treatment was further investigated. As shown in Fig. 4c, before post-printing treatment, the PVP-coated AgNWs were surrounded by insulated PEO matrix that hindered electrons transfer between neighboring nanowires, thus the resistance was relatively high. During the treatment, most of the PVP and PEO was dissolved in the DI water. As observed in high-magnification SEM images (Fig. 4d), after the treatment, some AgNW were in contact or even fused with each other; the direct contact and the fused junctions can contribute to decreasing the resistance of the printed AgNWs. During the post-printing treatment, especially water washing process, the ability of PVP to adsorb water shows a positive effect on the final electrical properties. The originally rigid nanowires can become softer at high humidity as reported recently^51–53^, which can facilitate the contact and fusion between AgNWs during the water washing process. In addition, thermal annealing can assist with the fusion between AgNWs too^32^. It is important to include the water washing in the post-printing treatment. Thermal annealing alone would require 200 °C and above, which can damage the PET substrate.

**Figure 5:**
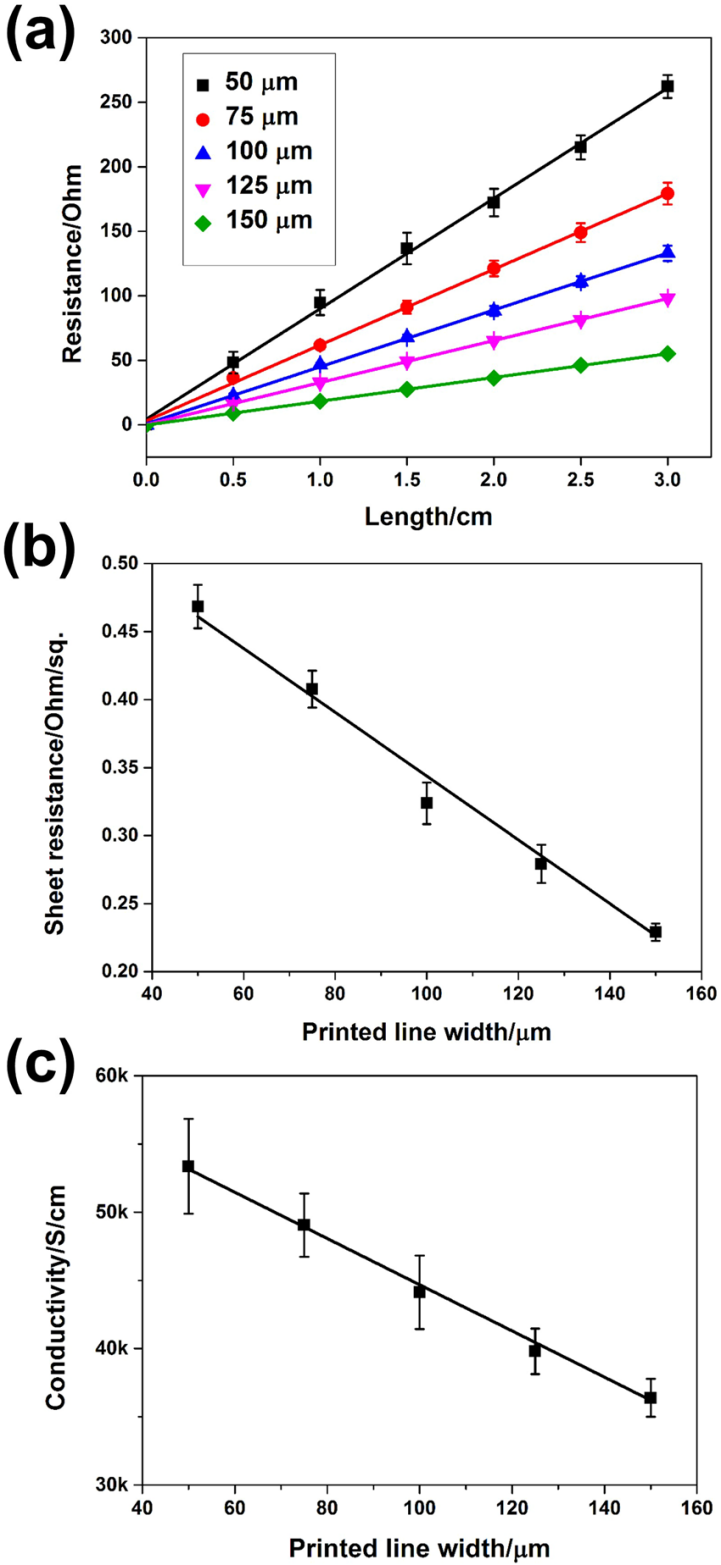
(**a**) Measured resistance of the gravure-printed AgNW lines at different length and with various line widths. (**b**) Calculated sheet resistance of the gravure-printed AgNW lines with various line widths. (**c**) Calculated conductivity of the gravure-printed AgNW lines with various line widths. At least three samples were tested for each line width.

To evaluate the electrical properties of the printed AgNWs, 3 cm-length AgNW lines with widths of 50, 75, 100, 125 and 150 μm were gravure-printed on a PET film and treated with thermal annealing and water washing. Figure 5a plots the measured resistance as a function of the length for all five AgNW lines. A clear linear relationship, with the correlation factors over 0.998 in all cases, can be seen, which indicates excellent uniformity of the printed AgNW lines of different widths. It can also been seen that the resistance decreases with increasing width of the AgNW lines. For example, the resistances were measured to be 262.2 ± 9.0, 179.2 ± 8.4, 130.1 ± 5.9, 98.1 ± 1.1 and 55.2 ± 0.4 Ω for the line widths of 50, 75, 100, 125 and 150 μm, respectively, with the corresponding sheet resistances calculated to be 0.468 ± 0.016, 0.408 ± 0.014, 0.324 ± 0.015, 0.279 ± 0.014 and 0.229 ± 0.006 Ω sq^−1^ (Fig. 5b). It can be seen that the sheet resistance of the printed AgNW lines decreases slightly as the line width increases. Brief explanation behind the calculation of sheet resistances can be seen in Supplementary Information, which was similar to calculation used for screen-printed^13^ and electrohydrodynamic-printed^54^ AgNW lines. The electrical conductivity was further calculated following the equation:

$${\rm{\sigma }}=\frac{{\rm{L}}}{{\rm{RA}}},$$

where *σ*, *R*, *L*, and *A* are conductivity, resistance, the length and the cross-sectional area of the AgNW lines, respectively. Based on the measured resistances and the line geometries, the conductivities were calculated to be (5.34 ± 0.35) × 10^4^, (4.91 ± 0.23) × 10^4^, (4.41 ± 0.27) × 10^4^, (3.98 ± 0.16) × 10^4^ and (3.64 ± 0.14) × 10^4^ S cm^−1^ for the line widths of 50, 75, 100, 125 and 150 μm, respectively. It can be seen that the electrical conductivity decreases slightly as the printed line width increases. A similar trend was observed in screen-printed AgNW lines, which was attributed to the presence of voids in the AgNW patterns with larger line widths^13^. In this work, however, only a few voids were observed and this trend is more likely due to the NW alignment. The AgNWs showed better alignment along the printing direction for the narrower line, as compared to the wider line where a significant proportion of AgNWs were randomly oriented (Fig. 3d,e). Better alignment can increase the electrical conductivity of the AgNWs. Table 1 compares the smallest line width and highest electrical conductivity of printed AgNW lines using several reported printing methods. It can be seen that by tailoring the ink properties and printing conditions, gravure printing can be used to print continuous AgNW lines with resolution as fine as ~50 μm and electrical conductivity as high as 5.34 × 10^4^ S cm^−1^. The combination of high resolution and high conductivity is among the best for all the printed AgNW lines.

**Table 1:** Comparison of NW diameter, NW length, (smallest) printed line width and (highest) electrical conductivity reported for AgNWs using different printing methods.

Printing method	NW diameter (nm)	NW Length (μm)	Line width (μm)	Conductivity (S•cm^−1^)	Ref.
Inkjet	55	2.2	1000	1 × 10^3^	^56^
EHD-jet	40 ± 7	20 ± 5	15	Non-conductive	^46^
Screen	25–35	15–25	50	4.67 × 10^4^	^13^
Gravure	50	22	230	1.81 × 10^4^	^25^
Gravure	80	15	50	5.34 × 10^4^	This work

**Figure 6:**
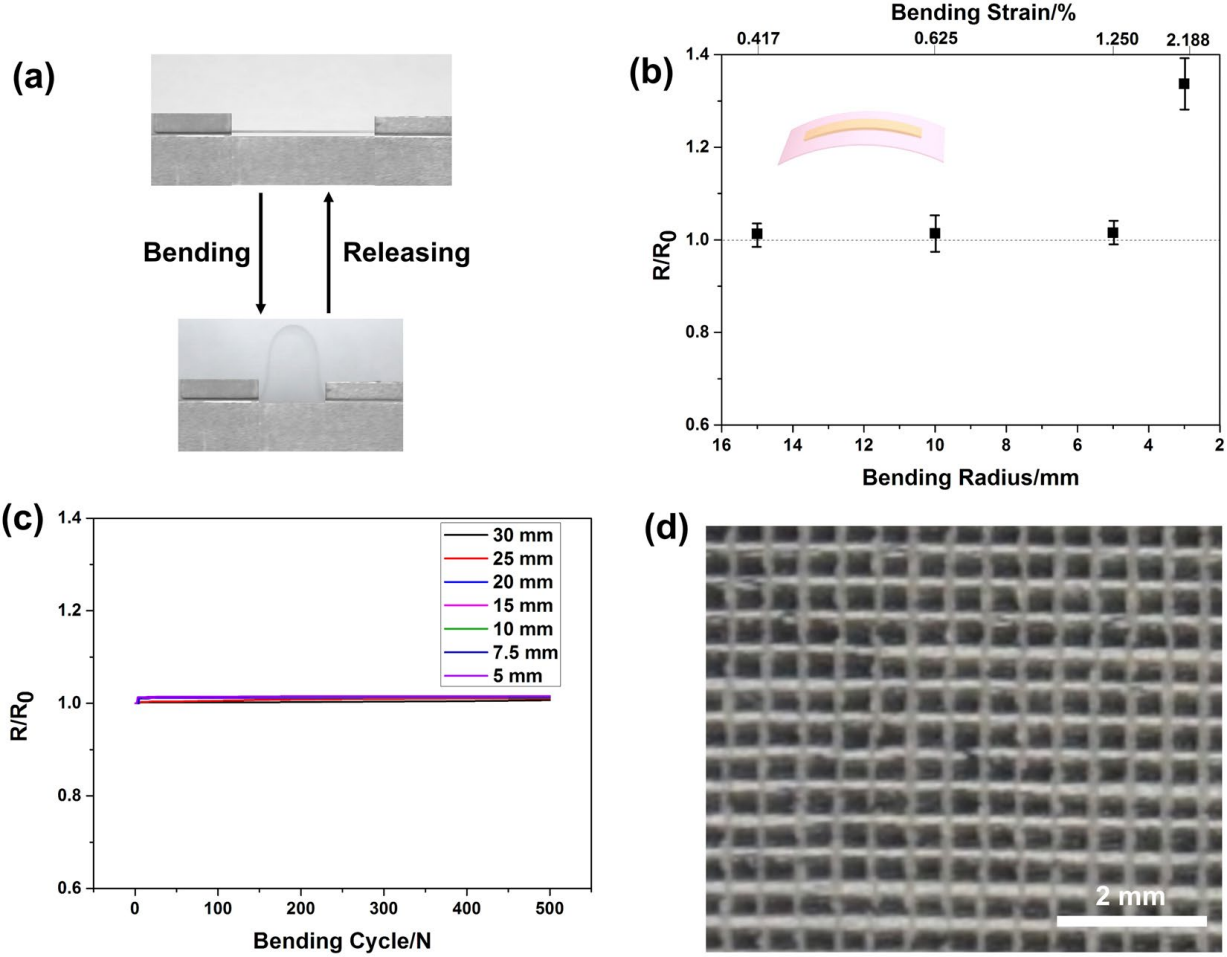
(**a**) Photograph of the bending test process. (**b**) Resistance changes of AgNW line as a function of tensile bending radius and bending strain. (**c**) Dynamic bending fatigue tests of AgNW line at the bending radii of 30, 25, 20, 15, 10, 7.5 and 5 mm, respectively. (**d**) Optical image of the printed 150 µm-width AgNW grid with line spacing of 500 µm.

In additional to being highly conductive, the printed AgNW lines exhibited robust mechanical responses under tensile bending condition, which is of critical relevance to flexible electronics. The bending test (Fig. 6a) results revealed that the gravure-printed AgNW line (100 µm in width) maintained a constant resistance until the bending radius reached 3 mm, or an equivalent bending strain of 2.08%, as shown in Fig. 6b. Figure 6c shows the cyclic bending test results for different bending radii (30, 25, 20, 15, 10, 7.5 and 5 mm). No obvious increase in resistance over 500 bending cycles to the smallest bending radius of ∼5.0 mm, which demonstrates the good mechanical flexibility and reliability of the gravure-printed AgNWs. As most of the PEO and PVP are removed from the printed AgNW lines, the poor adhesion of AgNWs and PET substrate can occur especially after about 50 times repeated cycles of adhering and peeling off (Fig. S6). On the other hand, the poor adhesion can make easier to transfer the printed AgNW lines onto other elastic substrates, *e.g.* by drop-casting liquid PDMS on top of the AgNW lines, which can be used as stretchable conductor^31–40^.

To demonstrate the general applicability of the developed gravure printing method, large-area and complicated AgNW patterns (*e.g*. lines, curves, Greek cross fractal patterns and grids) with different line widths and shapes were printed on PET films (Fig. S7). To print the AgNW grid, the lines parallel to the direction of printing were first printed. Then, by rotating the engraved plate by 90°, the orthogonal lines were printed. As shown in Fig. 6d, 150 μm-width lines were gravure-printed with line spacing of 0.5 mm. A well-known challenge for gravure printing is to print complicated patterns with arbitrary directions. As discussed above, to make sure the AgNW ink can fill in the trench, intaglio trenches were used in our experiments. While complicated patterns such as curves and Greek cross fractal patterns were printed, the well-known pickout effect in gravure printing might sometimes occur, where a small section of the printed patterns was different to transfer to the substrate^47^. This phenomenon warrants further development of gravure printing of long one-dimensional nanomaterials such as AgNWs.

## Conclusion

In summary, water-based AgNW inks were developed and gravure printed on flexible PET films. The AgNW ink, which contains a low solid content of 5.0 wt%, had a viscosity as high as 20.9 Pa s at 10 s^−1^ shear rate and appropriate rheological behavior suitable for gravure printing. Uniform and sharp-edged lines with resolution of 50 μm were obtained by gravure printing of the AgNW ink. Moreover, post-printing treatment with a low thermal annealing temperature of 150 °C and water washing was developed, which improved the electrical conductivity of the printed patterns to as high as 5.34 × 10^4^ S cm^−1^. In addition, gravure-printed large-area AgNW grids indicates that the integration of AgNWs with gravure printing holds promising potential for commercially relevant, highly scalable applications in printed and flexible electronics.

## Methods

### Materials

The reagents used in this study included silver nitrate (AgNO_3_), poly(vinylpyrrolidone) (PVP, K-30), sodium chloride (NaCl), ethylene glycol (EG), acetone, ethanol, Poly(ethylene oxide) (PEO, average M_v_ ~1,000,000). All reagents were of analytical grade and purchased from Sigma Aldrich. All chemicals were used as received without further purification.

### Synthesis of AgNWs

AgNWs were fabricated using a modified polyol reduction method^55^. PVP solution (50 mL 0.09 M in EG) was heated to 170 °C in a three-neck flask with stirring at 300 rpm for 1 h. NaCl solution (150 µL 0.1 M in EG) was then added and stirred for 10 min. AgNO_3_ solution (50 mL 0.06 M in EG) was subsequently added to the flask dropwise at a rate of 2.5 mL min^−1^. After AgNO_3_ solution was added to the flask, the oil bath reaction lasted another 20 min. The clear solution changed color to glistening gray, which indicated the formation of AgNWs. Then the solution was cooled to room temperature and was centrifuged at 2000 rpm for 10 min with acetone and ethanol, respectively, to remove solvent (EG), surfactant (PVP) and other impurities (*e.g.* small amount of Ag nanoparticles) in the supernatant.

### Preparation of AgNW Inks

Poly(ethylene oxide) (PEO, average Mv ~1,000,000) was purchased from Sigma Aldrich and used as received. 4 wt% PEO solution was prepared by dissolving PEO powder in a mixed solvent of ~50 wt% deionized water (DI water) and ~46 wt% ethanol. AgNWs with different weight content were added into the PEO solution and the as-prepared inks were stirred with magnetic stir bar the speed of 1000 rpm for 60 min to ensure uniform AgNW inks.

### Gravure Printing and Post-Printing Treatment of AgNW Patterns

Inverse direct gravure printing system, which used a flat printing plate to transfer patterns to a substrate on a roll, was set up in our lab. The flat printing plate, which contained engraved intaglio trenches, was fabricated by laser cutting of flat clear cast acrylic sheet (McMaster-Carr) using a laser cutter (VLS 6.60, Universal Laser Systems). Trenches with different width were obtained by controlling the power of the laser beam and the depths of all trenches were ~60 µm. A stainless steel cylinder with diameter of 25 mm was used as impression roller. Polyethylene terephthalate film (PET, MELINEX® 454, Dupont) with thickness of 125 μm was first treated with plasma cleaner (PDC-32G, Harris Plasma) for 1 min to enhance the hydrophilic property and then wrapped around the printing roller by an elastic double-sided tape. The angle between the printing plate and the doctor blade was fixed at 75^o^. The printing speed was ~1.5 mm·s^−1^. The printed AgNW patterns were first annealed on hot plate at 150 ^o^C for 2 min to evaporate the water and ethanol, followed by washing with DI water at 70 °C for 10 min to remove part of PVP and PEO. The thermal annealing and water washing process were repeated several times. Finally, the coating was annealed at 150 °C for 5 min to help fuse the AgNW junctions, and the final conductive AgNW patterns were obtained.

### Characterization

The morphologies of the as-synthesized AgNWs and the gravure-printed AgNWs lines were tested by field-emission scanning electron microscopy (SEM, FEI Quanta 3D FEG) operated at 5 kV. The transmission electron microscope (TEM) image of the as-synthesized AgNWs was obtained by field emission ultra-high resolution scanning transmission electron microscope (Field Emission STEM, JEOL 2010F). The structural characterization of the AgNW films was performed using X-ray diffraction (XRD, Rigaku SmartLab X-Ray Diffractometer) with Cu Kα radiation (λ = 0.1542 nm). Rheological behavior of the AgNW inks was measured using a Haake VT500 viscotester system. A pre-conditioning step at a shear rate of 0.1 s^−1^ for 10 s was applied before each test to assure uniformity of the fluids and all of the tests were done at room temperature (25 °C). Surface tension of the AgNW inks was tested by Ramé-Hart contact angle goniometer at room temperature (25 °C). The dimensions of the gravure-printed AgNW lines were obtained by using an optical microscope (Nikon Eclipse). Alignment of AgNWs in the gravure-printed line patterns was analyzed with ImageJ software and at least 100 AgNWs for each line were analyzed. Thickness of the gravure-printed AgNW patterns was measured by a Dektak profilometer. A Fluke 115 true RMS multimeter was used to measure the resistance of the printed AgNW lines. The mechanical stability test was performed using a lab-made bending test machine. Scotch tape was used to evaluate the adhesion of the printed AgNW line after post-printing treatment. A scotch tape was applied on the samples and after pressing them on the substrate by hands the tape was peeled off slowly.

## Electronic supplementary material

Supplementary Information
