# Supplementary material for: Gravure Printing of Water-based Silver Nanowire ink on Plastic Substrate for Flexible Electronics
Source: Sci Rep. 2018 Oct 11;8:15167. doi: 10.1038/s41598-018-33494-9 (PMC6181915; doi:10.1038/s41598-018-33494-9)
Supplement: Supplementary file 1 — Supplementary Information [file 41598_2018_33494_MOESM1_ESM.docx]

**Gravure Printing of Water-based Silver Nanowire ink on Plastic Substrate for Flexible Electronics**

Qijin Huang and Yong Zhu*

Department of Mechanical and Aerospace Engineering, North Carolina State University, Raleigh, North Carolina 27695-7910, USA
E-mail: [yong_zhu@ncsu.edu](mailto:yong_zhu@ncsu.edu)

**Calculation of sheet resistance of the gravure-printed AgNW lines**

According to the formula $\rho=R\frac{A}{L}$ and $s=\frac{\rho}{h}$ , where ρ is the electrical resistivity of the printed lines, A is the cross-sectional area of the printed lines, L is the length of the printed lines, *Rs* is the sheet resistance of the printed lines and h is the thickness of the printed lines. Thus, sheet resistance can be calculated by $Rs=R\frac{A}{L\times h}$. The length of the printed lines was 3 cm for all samples. The resistance of the printed lines can be measured by a multimeter, while the cross section and thickness of the printed lines can be measured by a Dektak profilometer. The cross section area and the thickness of the printed lines were integral area and average thickness, respectively.


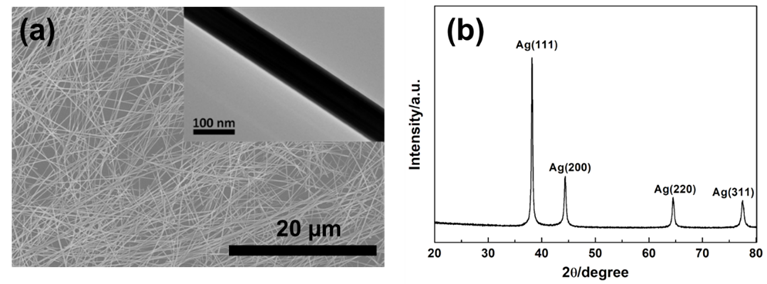


**Figure S1 (a)** SEM image and TEM image (inset of (a)) of the as-synthesized AgNWs. (b) XRD peaks of the as-synthesized AgNWs.


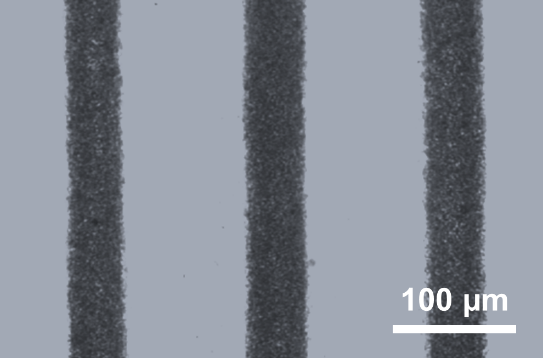


Figure S2 Optical image of three AgNW lines with the same width of 50 μm and the same spacing of 100 μm.


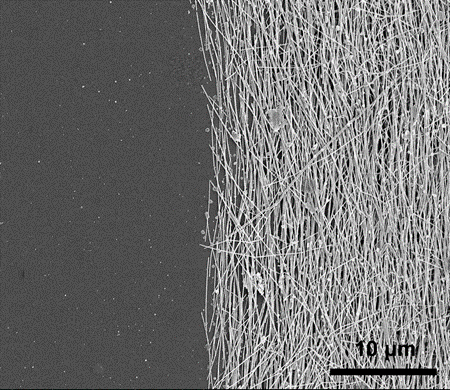


**Figure S3** SEM image of the edge of the printed line


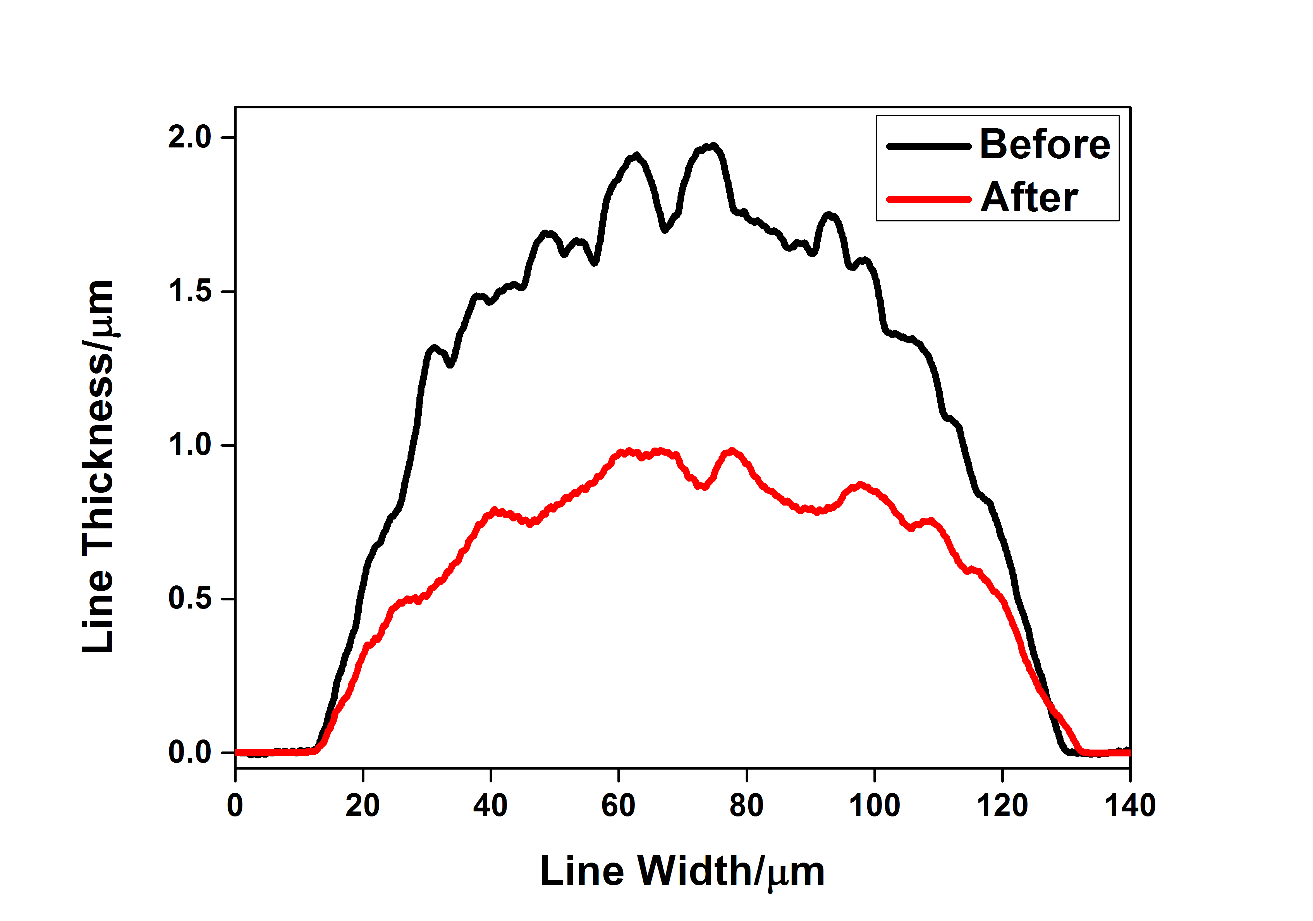


**Figure S4** Line thickness profiles measured by optical profilometry before treatment and after treatment cycles of six.

**
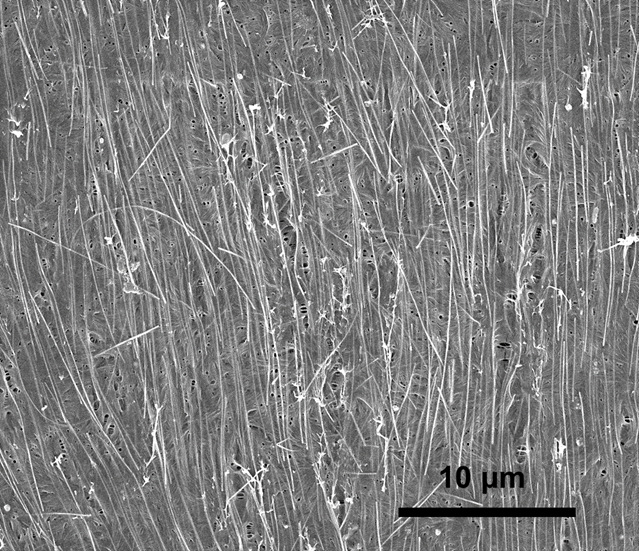
**

**Figure S5** SEM image of the line before post-printing treatment.


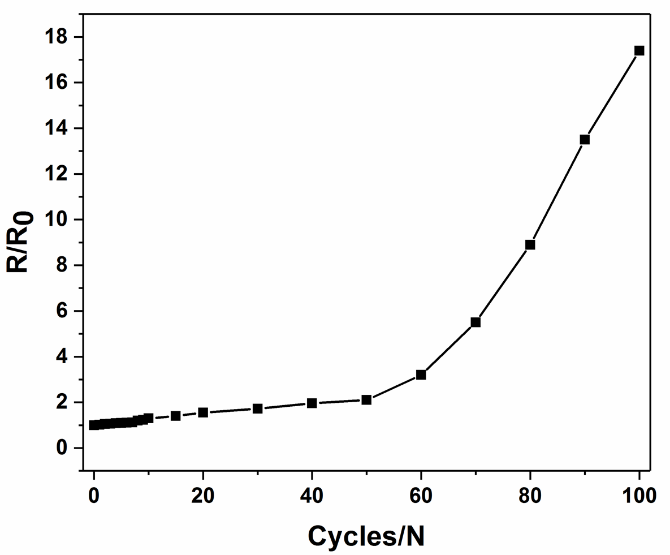


**Figure S6** Resistance change of the printed AgNW line after post-printing treatment versus cycles in adhesion test.


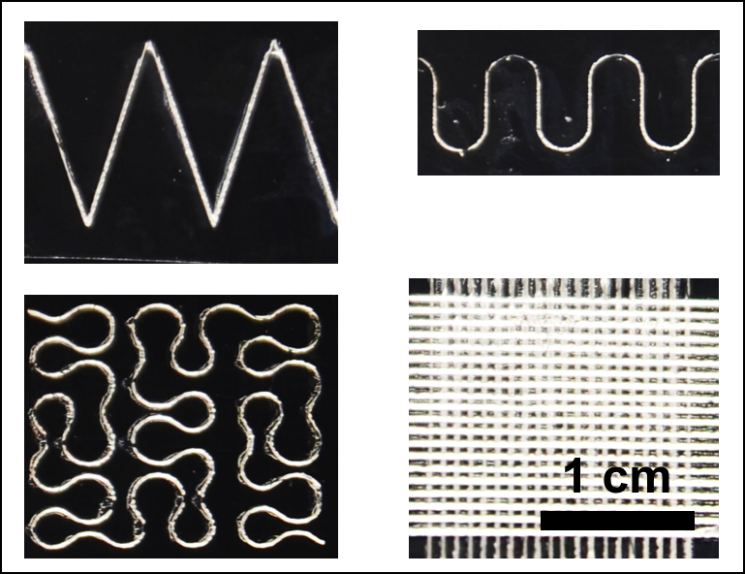


**Figure S7** Photographs of different gravure-printed AgNW patterns: lines, curves, Greek cross fractal pattern and AgNW grid.
